# Predictive value of HPSE for major adverse cardiovascular events in patients with ST-segment elevation myocardial infarction

**DOI:** 10.3389/fcvm.2026.1743115

**Published:** 2026-02-12

**Authors:** Wenyan Liu, Zonghu Jia, Bin Li, Yuhong Hu, Xuechao Tang, Zhen Li, Ruimin Chen, Zheng Wang, Shuo Yang, Zhenyu Niu, Shufang Han, Qun Jin

**Affiliations:** 1 Department of Cardiology, The 960th Hospital of the Joint Logistics Support Force of the Chinese people’s Liberation Army, Jinan, Shandong, China; 2 Graduate School, Shandong First Medical University & Shandong Academy of Medical Sciences, Jinan, Shandong, China

**Keywords:** heparanase, major adverse cardiovascular events, percutaneous coronary intervention, predictor, ST-segment elevation myocardial infarction

## Abstract

**Background:**

Although early mortality rates have decreased following reperfusion therapies such as percutaneous coronary intervention (PCI) in patients with ST-segment elevation myocardial infarction (STEMI), they remain at risk for major adverse cardiovascular events (MACE). Heparanase (HPSE) is an endogenous *β*-D-glucuronosidase and plays a key role in inflammation and vascular remodeling. This study investigated the predictive value of HPSE for MACE after STEMI.

**Methods:**

This prospective observational study included 207 consecutive STEMI patients who received primary PCI. Coronary blood was collected 10 min after balloon dilation. Coronary HPSE levels were measured by enzyme-linked immunosorbent assay (ELISA). Patients were followed for 6 months to record the occurrence of MACE.

**Results:**

During the 6-month follow-up period, 53 patients (25.6%) experienced MACE. Patients who experienced MACE had significantly higher coronary HPSE levels than those without (5.08 ± 1.27 ng/mL vs. 3.68 ± 1.20 ng/mL, *P* < 0.001). In multivariate Cox regression analysis, after adjusting for established risk factors, elevated HPSE levels remained an independent predictor of MACE (HR = 1.693, 95% CI: 1.323–2.165, *P* < 0.001). ROC analysis showed that a combined HPSE and GRACE model predicted MACE with an AUC of 0.849 (95% CI: 0.787-0.911, *P* < 0.001). Kaplan–Meier analysis revealed significantly lower MACE-free survival in the high HPSE group (log-rank *P* < 0.001).

**Conclusion:**

Coronary HPSE is an independent predictor of 6-month MACE in STEMI patients. It also enhances the predictive capability of the GRACE score. Because of this, HPSE serves as a promising biomarker that can help doctors assess risk more accurately and guide clinical decisions.

## 1 Introduction

Acute ST-segment elevation myocardial infarction (STEMI) is a severe cardiovascular disease (1, 2). It is a major focus in clinical practice because of high incidence and mortality rates (3). STEMI typically indicates cmplete coronary artery occlusion with the risk of transmural myocardial injury. Therefore, patients need immediate revascularization to save the heart muscle that is still alive (4). The main treatment to reopen the artery is percutaneous coronary intervention (PCI), which restores blood flow quickly and has greatly improved early survival (5, 6). But the process of restoring blood flow can itself cause additional damage. This is known as myocardial ischemia reperfusion injury (MIRI), which worsens cellular damage, triggers a body-wide inflammatory response, and leads to harmful changes in the heart's structure (7, 8). Because of this, patients still face a high risk of future problems even after successful treatment. They may experience major adverse cardiovascular events (MACE) including death from heart causes, reinfarction, severe angina, or heart failure (9, 10). Therefore we need strong biomarkers soon after PCI to help stratify patients by risk, guide personal secondary prevention plans and improve prognosis.

Heparanase (HPSE) is the only mammalian enzyme that cuts heparan sulfate (HS), which exerts both enzymatic and non-enzymatic signaling functions (11–13). In acute myocardial infarction (AMI), HPSE breaks down parts of the extracellular matrix and the endothelial glycocalyx (14), which contributes to atherosclerotic plaque destabilization and eventual rupture, leading to coronary blockage (15–18). After blood flow is restored, HPSE makes injury worse by driving oxidative stress, inflammation, dysregulated autophagy, and apoptosis (19–21). Together, these processes contribute to MIRI. Although HPSE is known to be harmful in heart attacks, it is still not clear whether it can help predict outcomes in STEMI patients after reperfusion therapy.

This study investigated the association between coronary HPSE concentration and MACE occurrence in STEMI patients undergoing primary PCI, and we hope this approach can improve long-term patient outcomes.

## 2 Materials and methods

### 2.1 Study design and population

This prospective observational cohort study was conducted at the Chest Pain Center of the 960th Hospital of the Joint Logistics Support Force of the Chinese People's Liberation Army. The study period spanned from September 2023 to February 2025, enrolling a total of 207 adult patients diagnosed with STEMI who successfully underwent PCI. Inclusion criteria were: (1) age ≥ 18 years; (2) Diagnosis of STEMI meeting the criteria of the *2023 ESC Guidelines for the management of acute coronary syndromes* (22); (3) Successful PCI with TIMI 3 flow in the infarct-related artery and final residual stenosis <20%; (4) Symptom-to-balloon time (STBT) ≤ 12 h. Exclusion criteria were as follows: (1) Failed PCI (final TIMI flow <3); (2) History of prior PCI or any cardiac surgery; (3) Presence of severe cardiovascular disease (including severe valvular heart disease, cardiomyopathy, or prior heart transplant), hepatic insufficiency (defined as serum alanine aminotransferase or aspartate aminotransferase levels >3 times the upper limit of normal), or renal insufficiency (defined as estimated glomerular filtration rate <30 mL/min/1.73 m²); (4) Recent use (within 2 weeks prior to admission) of medications that may significantly influence HPSE expression or release, including but not limited to: chemotherapeutic agents, systemic anti-inflammatory or immunomodulatory drugs, and long-term therapeutic-dose anticoagulants; (5) Major surgery or active bleeding within the past 4 weeks; (6) Presence of autoimmune disease, hematologic malignancy, active hepatitis, or any active solid tumor; (7) Acute or chronic infectious disease; (8) Pregnancy or lactation, psychiatric/neurological disorders, or inability to comply with study protocols; (9) Incomplete clinical data or loss to follow-up.

### 2.2 Clinical data

We collected complete baseline data for all patients. This included their demographic details, cardiovascular risk factors, vital signs at admission, and the GRACE risk score. We also recorded lab results from within 24 h of admission, and measured left ventricular ejection fraction (LVEF) using echocardiography.

### 2.3 Sample collection and HPSE concentration measurement

During the PCI procedure, after successful balloon angioplasty and reopening of the blocked artery, we took 5 mL of coronary blood from the guiding catheter at 10 min. The blood was quickly centrifuged to get serum. We then aliquoted the serum and stored it at −80°C in ultra-low temperature freezers for later batch testing. Coronary HPSE levels were measured using a commercial human HPSE ELISA kit from CUSABIO, China. We closely followed the manufacturer's instructions. The test showed good reproducibility, with both intra- and inter-assay coefficients of variation below 10%.

### 2.4 Endpoint events and follow-up

The main goal was to see if MACE happened within 6 months after PCI. MACE includes cardiovascular death, recurrent myocardial infarction, rehospitalization for heart failure, recurrent angina that needs hospital stay, or malignant arrhythmias. These arrhythmias included ventricular tachycardia, ventricular fibrillation, or high-grade atrioventricular block. We followed all patients at 1, 3, and 6 months after they left the hospital. We used telephone interviews and reviewed outpatient or hospital records to accurately record if any endpoint events occurred and when they happened.

### 2.5 Statistical analysis

All statistical analyses were done with SPSS Statistics (Version 29.0.2.0). Continuous variables that were normally distributed are shown as mean ± standard deviation and were compared between groups with the independent samples t-test. For continuous variables that were not normally distributed, we show median and interquartile range and used the Mann–Whitney U test to compare them. Categorical variables are given as numbers and percentages and were compared using the chi-square test or Fisher's exact test when needed.

We used univariate and multivariate Cox proportional hazards regression models to study the link between HPSE levels and the risk of MACE. To show the independent and added predictive value of HPSE, we built three multivariate models: Model 1 adjusted for traditional clinical factors, Model 2 included the GRACE score plus HPSE, and Model 3 was a full model with all significant covariates. The results are given as hazard ratios (HR) with 95% confidence intervals (CI).

We evaluated how well HPSE, the GRACE score, and their combination could predict MACE using receiver operating characteristic (ROC) curve analysis. We report the area under the curve (AUC) and the sensitivity and specificity at the best cutoff value, each with 95% CIs. We also plotted survival curves with the Kaplan–Meier method and compared MACE-free survival between groups with the log-rank test. To further quantify the incremental predictive value of HPSE beyond the GRACE score, we calculated the net reclassification improvement (NRI) and integrated discrimination improvement (IDI). A two-sided *P* value below 0.05 was considered statistically significant for all analyses, with the exception that a one-sided test was specifically applied for the IDI.

### 2.6 Ethical approval and informed consent

The study was conducted in accordance with the Declaration of Helsinki and approved by the Institutional Review Board of The 960th Hospital of the Joint Logistics Support Force of the Chinese People's Liberation Army (protocol code 2022-163 and date of approval 19 January 2022). Informed consent was obtained from all subjects involved in this study.

## 3 Results

### 3.1 Clinical outcomes at 6-month follow-up

The study looked at 207 STEMI patients who had a successful PCI. During the 6-month follow-up, 53 patients (25.6%) had MACE. This included heart failure (*n* = 20), recurrent angina (*n* = 21), recurrent myocardial infarction (*n* = 7), malignant arrhythmia (*n* = 3), and cardiovascular death (*n* = 2). These results are shown in Table 1.

**Table 1:** Major adverse cardiovascular events (MACE) within 6 months after primary PCI.

Endpoint event	Number of patients, *n* (%)
Primary composite endpoint: MACE	53 (25.6)
Components of MACE	
Heart failure	20 (37.7)
Severe angina	21 (39.6)
Recurrent myocardial infarction	7 (13.2)
Malignant arrhythmia	3 (5.7)
Cardiovascular death	2 (3.8)

The percentage for the primary endpoint (25.6%) is calculated from the total patient population (*N* = 207). The percentages for the individual MACE components are calculated from the total number of patients who experienced a MACE (*n* = 53).

### 3.2 Baseline characteristics of the MACE group and non-MACE group

**Figure 1:**
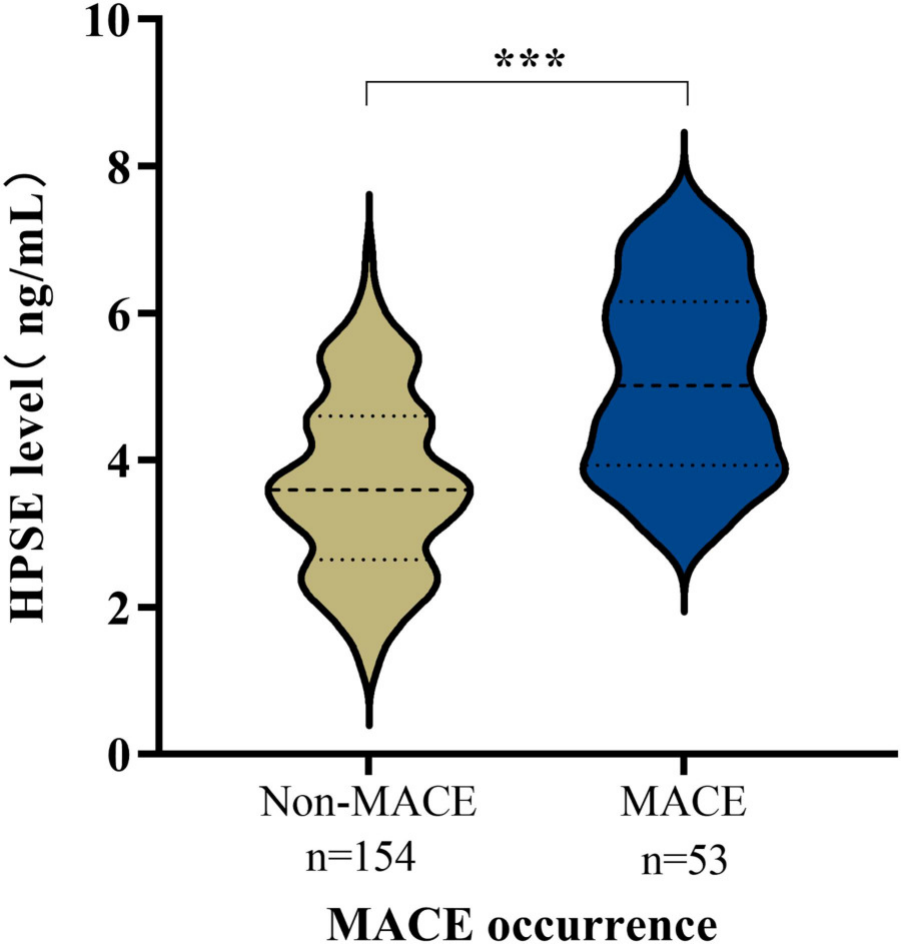
Comparison of coronary blood HPSE levels between STEMI patients with and without MACE.

Patients who experienced MACE presented with significantly worse clinical profiles at baseline compared to those without MACE. Specifically, the MACE group had lower left ventricular ejection fraction (LVEF), reduced estimated glomerular filtration rate (eGFR), elevated cardiac troponin I (cTnI) levels, and higher GRACE risk scores (all *P* < 0.001). Crucially, and as a central finding of this study, coronary HPSE concentrations were also significantly elevated in the MACE group (5.08 ± 1.27 ng/mL vs. 3.68 ± 1.20 ng/mL, *P* < 0.001; Figure 1). No other significant differences in baseline characteristics were observed between the two groups (all *P* > 0.05). As detailed in Table 2.

**Table 2:** Baseline characteristics of STEMI patients stratified by the occurrence of MACE.

Characteristics	Over all (*n* = 207)	Non-MACE group (*n* = 154)	MACE group (*n* = 53)	*P*-value
Demographics				
Age, years	58.95 ± 10.84	58.12 ± 11.28	61.38 ± 9.10	0.059
Male, *n* (%)	135 (65.22)	99 (64.29)	36 (67.92)	0.631
BMI, kg/m²	26.34 ± 2.72	26.16 ± 2.81	26.86 ± 2.36	0.105
Smoking history, *n* (%)	130 (62.80)	95 (61.69)	35 (66.04)	0.572
Medical history				
Diabetes	85 (41.06)	65 (42.21)	20 (37.74)	0.568
Hypertension	129 (62.32)	99 (64.29)	30 (56.60)	0.320
Laboratory parameters				
LDL-C, mmol/L	2.98 ± 0.76	2.93 ± 0.74	3.11 ± 0.79	0.134
AST, U/L	40 (25, 53)	38.5 (24, 53)	40 (30, 53)	0.357
ALT, U/L	31 (21, 45)	29 (21, 44)	33 (24, 45)	0.081
eGFR, mL/min/1.73 m²	111.60 (95.8, 128.5)	116.30 (101.83, 131.43)	97.80 (85.15, 116.45)	**<0**.**001**
NT-proBNP, pg/mL	591.60 (279.20, 1,407.60)	509.82 (273.50, 1,225.45)	820.40 (290.40, 1,702.10)	0.095
cTnI, ng/mL	46.48 (28.50, 90.00)	40.76 (24.67, 72.86)	76.43 (46.02, 121.85)	**<0**.**001**
Clinical parameters				
Systolic BP, mmHg	122.53 ± 13.64	123.07 ± 14.19	129.94 ± 11.86	0.328
Diastolic BP, mmHg	77.88 ± 10.60	78.64 ± 11.14	75.70 ± 8.56	0.082
LVEF, %	52 (47, 56)	53 (49, 56)	47 (42, 54)	<**0**.**001**
GRACE score	144.48 ± 26.01	137.22 ± 21.63	165.58 ± 26.36	<**0**.**001**
Coronary artery disease characteristics				
Culprit artery				0.422
LM	7 (3.38)	6 (3.90)	1 (1.89)	
LAD	90 (43.48)	62 (40.26)	28 (52.83)	
LCX	34 (16.43)	26 (16.88)	8 (15.09)	
RCA	76 (36.71)	60 (38.96)	16 (30.19)	
Procedural characteristics				
Symptom-to-balloon time, hours	6 (4, 10)	5.5 (4, 10)	6 (3, 8)	0.223
Coronary HPSE, ng/mL	4.04 ± 1.36	3.684 ± 1.204	5.08 ± 1.27	**<0**.**001**

ALT, alanine aminotransferase; AST, aspartate aminotransferase; BMI, body mass index; BP, blood pressure; CI, confidence interval; cTnI, cardiac troponin I; eGFR, estimated glomerular filtration rate; HPSE, heparanase; HR, hazard ratio; LAD, left anterior descending artery; LCX, left circumflex artery; LDL-C, low-density lipoprotein cholesterol; LM, left main coronary artery; LVEF, left ventricular ejection fraction; MACE, major adverse cardiovascular events; NT-proBNP, B-type Natriuretic Peptide Prohormone; RCA, right coronary artery; STEMI, ST-segment elevation myocardial infarction.

A two-sided *P*-value < 0.05 was considered statistically significant and is highlighted in bold.

### 3.3 Univariate Cox regression analysis of MACE predictors

Univariate Cox regression identified several significant predictors of MACE (Table 3). Reduced LVEF and lower eGFR emerged as risk factors significantly associated with increased MACE risk. Similarly, elevated cTnI, higher GRACE score, and increased coronary HPSE concentration were also identified as significant risk factors, all showing strong associations (all *P* < 0.001).

**Table 3:** Univariate Cox proportional hazards analysis for MACE in STEMI patients.

Variable	HR (95% CI)	*p*-value
Age (per 10-year increase)	1.025 (1.000–1.051)	0.053
Male (vs. female)	1.182 (0.664–2.105)	0.570
BMI (per 5 kg/m² increase)	1.474 (0.930–2.337)	0.099
Smoking history (yes vs. no)	1.212 (0.686–2.139)	0.508
Medical history		
Diabetes (yes vs. no)	0.870 (0.499–1.516)	0.622
Hypertension (yes vs. no)	0.780 (0.453–1.343)	0.370
Laboratory parameters		
LDL-C (per 1 mmol/L increase)	1.302 (0.933–1.817)	0.121
AST (per 10 U/L increase)	1.118 (0.965–1.296)	0.137
ALT (per 10 U/L increase)	1.074 (0.953–1.210)	0.244
eGFR (per 20 mL/min/1.73 m² decrease)	2.300 (1.643–3.220)	**<0**.**001**
NT-proBNP (per 100 pg/mL increase)	1.027 (0.994–1.062)	0.111
cTnI (per 10 ng/mL increase)	1.153 (1.095–1.215)	**<0**.**001**
Clinical parameters		
Systolic BP (per 20 mmHg decrease)	1.233 (0.824–1.845)	0.308
Diastolic BP (per 10 mmHg decrease)	1.267 (0.971–1.653)	0.081
LVEF (per 5% decrease)	2.048 (1.591–2.636)	**<0**.**001**
GRACE score (per 30-point increase)	3.362 (2.453–4.607)	**<0**.**001**
Study variable		
Coronary HPSE (per 1 ng/mL increase)	2.252 (1.790–2.833)	**<0**.**001**

Abbreviations and statistical methods are as defined in Table 2. A *p*-value < 0.05 was considered statistically significant and is highlighted in bold.

### 3.4 Multivariate Cox regression analysis of predictors for MACE

Multivariable Cox regression analyses were conducted using three distinct models (Table 4). Model 1, which included established clinical factors, identified decreased LVEF (HR = 1.703), decreased eGFR (HR = 1.852), and elevated cTnI (HR = 1.134) as independent predictors of MACE (all *P* < 0.001). Model 2, which incorporated the GRACE score and coronary HPSE, confirmed both as significant independent predictors (HR = 2.596 and HR = 1.957, respectively; all *P* < 0.001). Most importantly, in the combined Model 3, which included all these variables, coronary HPSE concentration remained an independent predictor (HR = 1.693, 95% CI: 1.323–2.165, *P* < 0.001), alongside LVEF, eGFR, and cTnI.

**Table 4:** Multivariate Cox regression analysis of predictors for MACE.

Variable	Model 1: Established clinical factors	Model 2: GRACE + HPSE	Model 3: Combined model
	HR (95% CI)	HR (95% CI)	HR (95% CI)
Coronary HPSE (per 1 ng/mL increase)	–	1.957 (1.540–2.488)**	1.693 (1.323–2.165)***
GRACE score (per 30-point increase)	–	2.596 (1.888–3.569)**	–
LVEF (per 5% decrease)	1.703 (1.325–2.189)***	–	1.405 (1.071–1.842)*
eGFR (per 20 mL/min/1.73 m² decrease)	1.852 (1.329–2.582)***	–	1.726 (1.244–2.394)**
cTnI (per 10 ng/mL)	1.134 (1.076–1.194)***	–	1.092 (1.032–1.156)**

Abbreviations as in Table 2. Model 1: Adjusted for established clinical predictors (LVEF, eGFR, cTnI); Model 2: Adjusted for GRACE score and coronary HPSE level; Model 3: Adjusted for coronary HPSE level and established clinical predictors (LVEF, eGFR, cTnI).

* *p* < 0.05.

** *p* < 0.01.

*** *p* < 0.001.

### 3.5 Predictive performance of HPSE and GRACE scores for MACE

**Figure 2:**
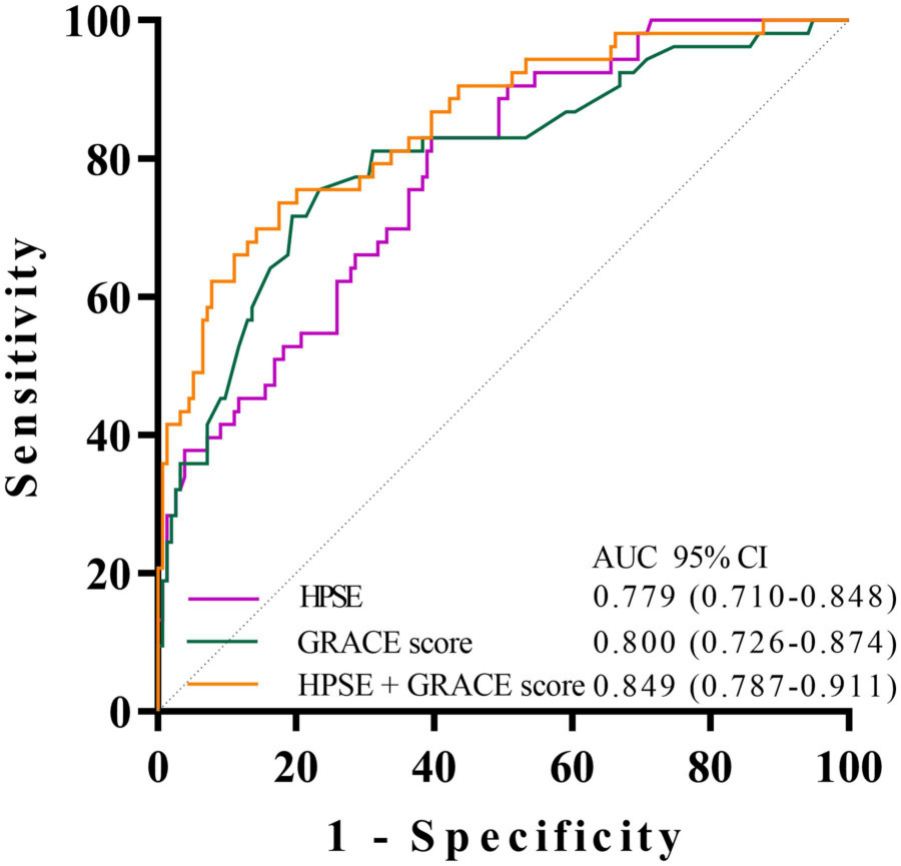
ROC curves for predicting MACE by coronary HPSE, GRACE score, and their combination.

ROC analysis showed that coronary HPSE level can predict MACE, with an AUC of 0.779. At the best cutoff value of 3.813 ng/mL, the sensitivity was 60.4% and the specificity was 83.0%. The GRACE score had similar predictive power, with an AUC of 0.800. Importantly, a combined model using both HPSE and GRACE score gave better results. Its AUC was 0.849 (95% CI: 0.787–0.911, *P* < 0.001). These findings are shown in Figure 2 and Table 5.

**Table 5:** ROC curve analysis of coronary HPSE, GRACE score, and their combination for predicting MACE.

Variable	Sensitivity (%)	Specificity (%)	AUC（95% CI）	*P* value	Cut off
HPSE	0.604	0.830	0.779 (0.710–0.848)	< 0.001	3.813 ng/mL
GRACE score	0.805	0.717	0.800 (0.726–0.874)	< 0.001	–
HPSE + GRACE score	0.825	0.717	0.849 (0.787, 0.911)	< 0.001	–

AUC, area under the curve; other abbreviations as in Table 2.

The *P* value tests the hypothesis that the AUC is greater than 0.5. The optimal cut-off value for HPSE (3.813 ng/mL) was determined by maximizing the Youden index (Sensitivity + Specificity − 1).

The incremental value of coronary HPSE was further assessed using NRI and IDI. The addition of HPSE to the GRACE model resulted in a statistically significant improvement in risk reclassification: the continuous NRI was 0.52 (95% CI: 0.25–0.81, *p* = 0.002), and the IDI was 0.015 (95% CI: 0.003–0.028, one-sided *p* = 0.008). These findings confirm that HPSE provides substantial incremental prognostic information beyond the GRACE score alone.

### 3.6 Survival analysis without MACE based on HPSE levels

**Figure 3:**
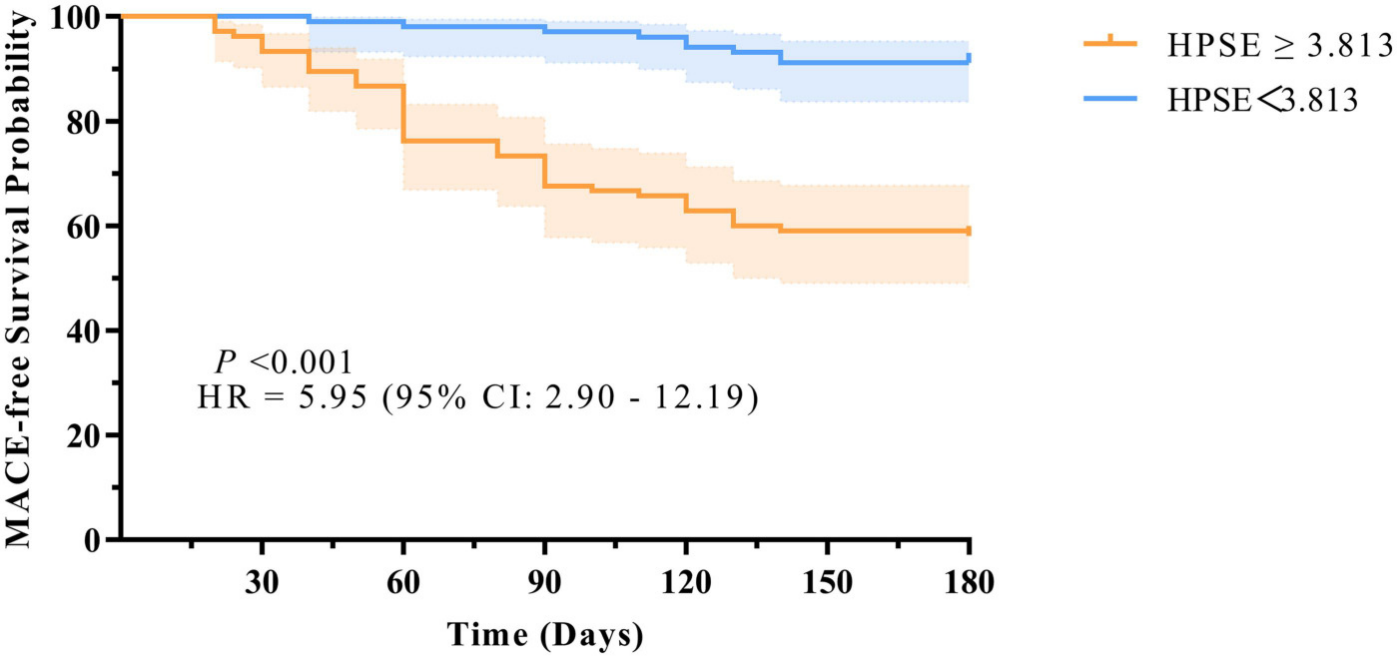
Kaplan–Meier curves for MACE-free survival stratified by coronary HPSE level.

We used the ROC-derived cutoff of 3.813 ng/mL to divide patients into two groups: high HPSE (≥3.813 ng/mL) and low HPSE (<3.813 ng/mL). Kaplan–Meier analysis showed that patients with high HPSE levels had significantly worse MACE-free survival (log-rank *P* < 0.001). Compared to the low HPSE group, the high HPSE group had a much higher risk of MACE, with a hazard ratio of 5.95 (95% CI: 2.90–12.19, *P* < 0.001). These results are shown in Figure 3.

### 3.7 Correlation between HPSE and inflammatory and myocardial injury markers

**Figure 4:**
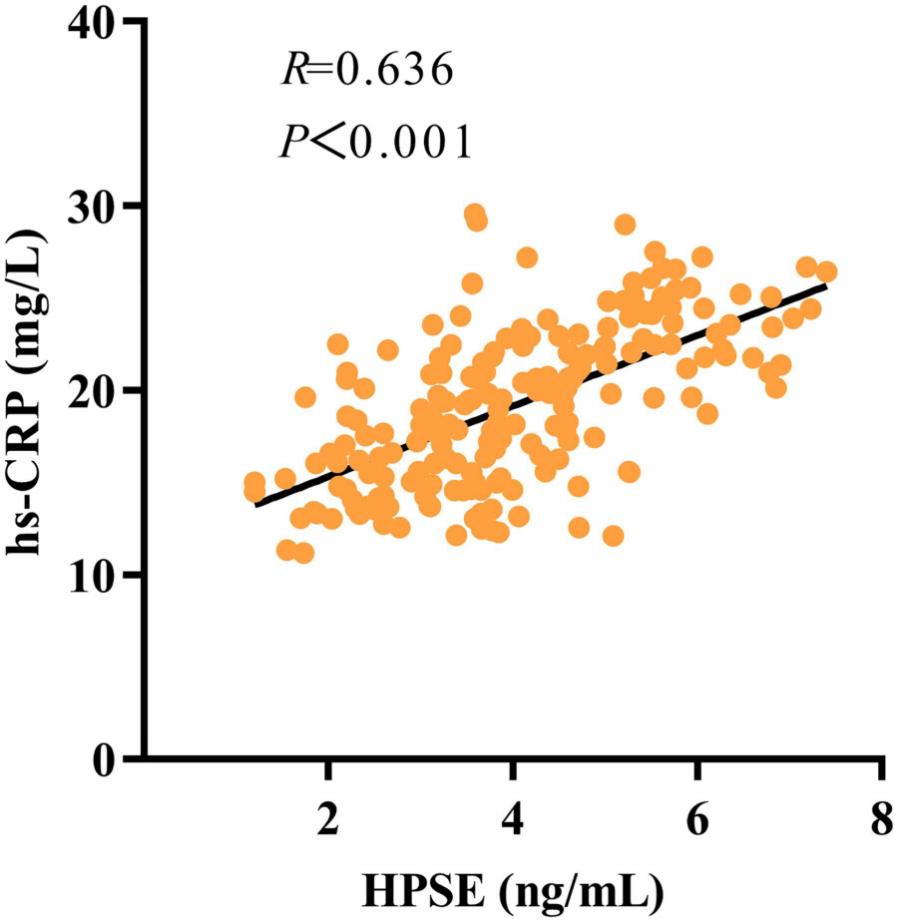
Scatter plot depicting the correlation between HPSE levels and hs-CRP levels.

**Figure 5:**
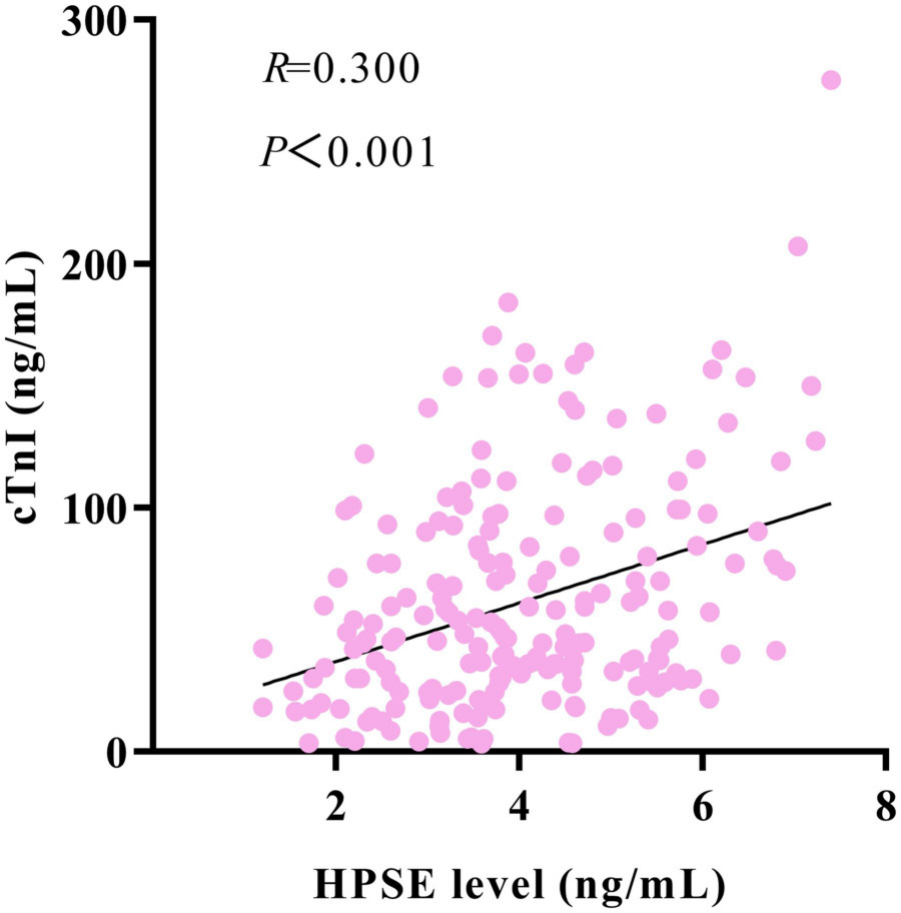
Scatter plot showing the correlation between HPSE levels and cTnI levels.

Spearman correlation analysis showed a strong positive link between coronary HPSE and hs-CRP levels (r = 0.636, *P* < 0.001). HPSE also had a moderate positive correlation with cTnI (r = 0.300, *P* < 0.001). Both correlations were statistically significant, as shown in Figures 4, 5.

## 4 Discussion

### 4.1 HPSE in the context of cardiovascular research

Accumulating evidence has highlighted the role of HPSE in the pathogenesis and progression of cardiovascular diseases. In preclinical studies, HPSE gene knockout in ApoE^−^/^−^ mouse models has been shown to attenuate atherosclerosis and enhance plaque stability, suggesting that HPSE promotes atherosclerotic progression and plaque destabilization (14). Clinical research further supports this mechanism. For example, Gurbuz et al. (23) reported that elevated peripheral blood HPSE levels before angiography in STEMI patients undergoing emergency PCI were significantly associated with increased thrombus burden and the no-reflow phenomenon, underscoring the pathophysiological role of HPSE in acute coronary events. Furthermore, Ma et al. (24) through bioinformatic analysis, found significant upregulation of the HPSE gene in MIRI and confirmed elevated HPSE expression in myocardial tissue of MIRI mice, indicating its involvement in reperfusion injury.

However, although these studies reveal potential mechanisms of HPSE in atherosclerosis and acute myocardial injury, the prognostic value of local HPSE concentration in the infarct-related artery after reperfusion therapy for patient outcomes remained unclear. Our study addresses this critical gap and demonstrates that coronary HPSE level is an independent predictor of short-term MACE in STEMI patients following primary PCI. Moreover, combining HPSE with the GRACE risk score significantly enhances early clinical risk stratification.

### 4.2 HPSE as a potential pleiotropic mediator of adverse outcomes

HPSE is not just a passive biomarker but also appears to be an active upstream regulator involved in post-ischemic myocardial injury, and may thereby influence the entire process of ischemia and reperfusion injury through several key mechanisms.

#### 4.2.1 HPSE is a key catalyst that may exacerbate local and systemic inflammatory responses

A strong positive correlation was found between coronary HPSE levels and hs-CRP (r = 0.636, *P* < 0.001). This shows that HPSE is associated with a proinflammatory role in STEMI. Mechanistically, HPSE is known to cut HS side chains and release bound proinflammatory factors such as IL-1β, IL-6, and TNF-α (11, 18). As a result, local inflammation and leukocyte recruitment increase. At the same time, HS fragments are generated. These fragments can act as damage-associated molecular patterns (DAMPs) and activate the TLR4-NF-κB signalling pathway (25). This could perpetuate a cycle of inflammation that maintains a persistent inflammatory state after infarction. In this study, hs-CRP did not show significant predictive value. In contrast, HPSE demonstrated strong predictive value. This difference highlights the potential unique role of HPSE. Hs-CRP indicates a general and delayed systemic inflammatory response. HPSE may reflect upstream and active drivers of inflammation. Therefore, HPSE could offer greater specificity and earlier predictive information. This means that it may enable the earlier identification of high-risk STEMI patients in a state of intense early inflammatory activation. This identification helps to categorize such patients into a higher risk stratum, thereby facilitating closer monitoring and more aggressive early intervention.

#### 4.2.2 HPSE may contribute to microcirculatory dysfunction

Microcirculatory impairment following coronary recanalization significantly affects outcomes in STEMI patients (26, 27). HPSE may play a key role in this process by degrading the endothelial glycocalyx (25). The glycocalyx is essential for maintaining vascular integrity, which helps to prevent leukocyte adhesion and inhibits thrombus formation (28). Damage to the glycocalyx can increase vascular permeability. This results in tissue edema and promotes aggregation of leukocytes and platelets. Consequently, these changes may lead to the no-reflow phenomenon (29). In our study, elevated HPSE levels were associated with adverse clinical events such as recurrent angina and reinfarction. This is consistent with the proposed mechanism. Furthermore, HPSE induces the formation of neutrophil extracellular traps (NETs), which can obstruct microvessels and increase thrombosis risk, thereby potentially further impairing tissue perfusion (19).

#### 4.2.3 HPSE may promote adverse ventricular remodeling

HPSE breaks down HS in the extracellular matrix of the heart. This loss of HS reduces structural support for cardiomyocytes. As a result, the heart may undergo adverse ventricular remodeling. This means the infarct grows larger, the ventricular wall gets thinner, and the heart chamber enlarges. These changes were observed in association with adverse clinical data, such as lower LVEF (HR = 2.048) and a high rate of heart failure (20/53). In addition, HPSE releases and activates pro-fibrotic factors such as TGF-β. These factors are normally stored in the matrix. Once released, they disturb normal collagen deposition leading to poorly organized and mechanically weak scar tissue. Together, these actions are likely to accelerate the progression to heart failure (30).

### 4.3 Association between HPSE and clinical indicators with incremental predictive value

We found a modest but significant positive correlation between HPSE and the myocardial necrosis marker cTnI (r = 0.300). This suggests that HPSE levels may be partly related to the initial infarct size. We included both factors in a multivariable analysis. Even after this adjustment, HPSE still independently predicted outcomes. This indicates that HPSE provides prognostic information that is not just about the initial necrosis extent. Its added predictive value likely comes from the potential role of HPSE in secondary injury mechanisms. These could include strong inflammation, microcirculatory dysfunction, and extracellular matrix degradation, as discussed earlier. Thus, HPSE has distinct clinical importance as a multifactorial driver of pathology after infarction.

### 4.4 Clinical implications and future perspectives

The present findings are important for clinical use. First, HPSE offers a new way to assess risk. We combined HPSE with the GRACE score, and this model predicted risk better, with an AUC of 0.849. This means that when we add key disease-related molecules to standard clinical scores, we can better find high-risk patients early. As a result, these patients could receive stronger treatment and closer monitoring. Crucially, the statistically significant improvements in both the NRI (0.52) and the IDI (0.015) provide direct quantitative evidence that HPSE not only enhances the model's overall discriminative capacity but also meaningfully improves the correct reclassification of individuals into clinically actionable risk categories.

More importantly, HPSE represents a potential therapeutic target. Current treatment for STEMI focuses on rapid revascularization, but there are few effective ways to prevent reperfusion injury. Given that HPSE may regulate multiple injury pathways upstream, it could be a suitable target for intervention. This study provides a rationale for exploring the use of HPSE inhibitors, first developed for cancer, in heart disease. Future research should test whether blocking HPSE during reperfusion can reduce inflammation and microvascular damage. This may help protect the heart muscle and improve long-term patient outcomes.

### 4.5 Limitations

This study has some limitations. First, it was done at one hospital with a medium number of patients. This may make the results less general and could cause selection bias. Second, the early coronary blood sampling protocol, while enabling direct assessment of HPSE at the site of the culprit lesion, limits broader clinical applicability. Future studies are needed to determine whether HPSE measured at more convenient time points or in peripheral venous blood retains robust predictive accuracy, which is crucial for wider clinical translation. Third, the follow-up time was only six months. This is enough to check short-term effects, but longer studies are needed to learn about the long-term role of HPSE. Lastly, as an observational study, our findings demonstrate an association but cannot establish a causal relationship between HPSE levels and clinical outcomes. Furthermore, the exact molecular pathways through which HPSE exerts its effect require further mechanistic investigation.

## 5 Conclusions

In summary, coronary HPSE level is a new and independent predictor of MACE in STEMI patients after PCI. HPSE is linked to multiple pathways that are associated with poor outcomes, including MIRI, inflammatory activation, microvascular dysfunction, and harmful heart remodeling. Combining HPSE with the GRACE score improves risk assessment in these patients. Moreover, because HPSE plays a key role in disease development, and its mechanistic roles suggest it could be a promising candidate for future therapeutic investigation.

## Data Availability

The raw data supporting the conclusions of this article will be made available by the authors, without undue reservation.
